# Supplementary material for: A Cross-Sectional Study of Potential Antimicrobial Resistance and Ecology in Gastrointestinal and Oral Microbial Communities of Young Normoweight Pakistani Individuals
Source: Microorganisms. 2023 Jan 20;11(2):279. doi: 10.3390/microorganisms11020279 (PMC9965051; doi:10.3390/microorganisms11020279)
Supplement: Supplementary file 1 [file microorganisms-11-00279-s001.zip › microorganisms-2170208-supplementary.pdf]

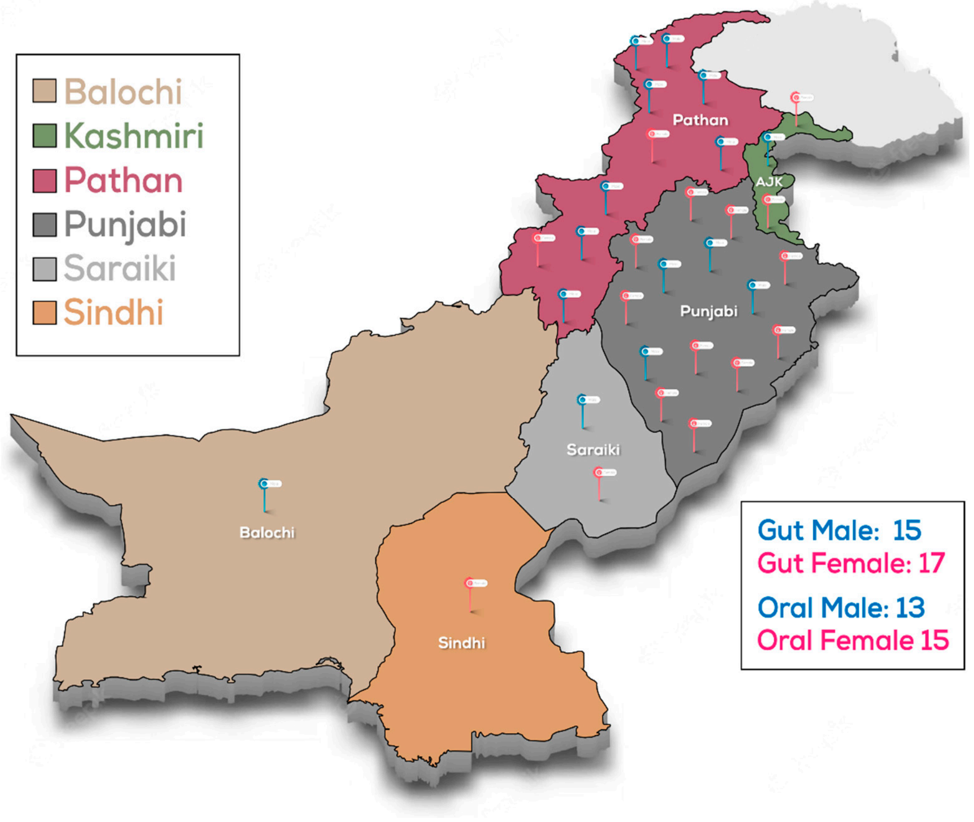

Supplementary Figure S1. Map highlighting the Ethnicities of participants: Self-reported ethnicities of healthy individuals at the time of sample collection, which is primarily based on their place of birth (highlighted on the map). The majority of these samples were paired as the individuals provided both gut and oral samples.

Supplementary Table S1. Summary statistics of the samples analyzed. A total of 60 samples were assigned to four major groups: Gut Male, Gut Female, Oral Male and Oral Female. Summary statistics, including median and Interquartile Range (IQR) of continuous variables such as *Age* and *BMI,* is given.

	**Groups**	**Total Samples (n)**	**Ethnic Groups (n)**	**Age (Years)** ***18-40***		**BMI (Kg/m2)** ***18-25***		**Smoking (n)**		**Antibiotics Usage (Past 3 months) (n)**		**Fresh Fruits (n)**		**Junk food (n)**		**Sources of drinking water (n)**
				**Median**	**IQR**	**Median**	**IQR**	**Yes**	**No**	**Yes**	**No**	**Yes**	**No**	**Yes**	**No**	
**1**	Gut Male	15	Balochi:1<br>Kashmiri:1<br>Pathan:8<br>Punjabi:4<br>Saraiki:1	23	19.50-25.50	22.4	20.50-24.40	5	10	2	13	4	11	13	2	Bottled: 2<br>Filtered: 4<br>Mineral: 3<br>Tap: 6
**2**	Gut Female	17	Kashmiri:2<br>Pathan:2<br>Punjabi:10<br>Saraiki:2<br>Sindhi:1	24	20-24	22.3	21.3-23.9	3	14	5	12	7	10	9	8	Bottled:1<br>Filtered:12<br>Mineral:2<br>Tap:2

**3**	Oral Male	13	Balochi:1<br>Kashmiri:1<br>Pathan:8<br>Punjabi:2<br>Saraiki:1	23	19-25	21	20.10-24.70	4	9	2	11	3	10	11	2	Bottled: 2<br>Filtered: 3<br>Mineral: 2<br>Tap: 6
**4**	Oral Female	15	Kashmiri:2<br>Pathan:2<br>Punjabi:9<br>Saraiki:2	24	22-24.50	21.9	21.10-23.85	3	12	4	11	6	9	7	8	Bottled: 1<br>Filtered: 10<br>Mineral: 2<br>Tap: 2

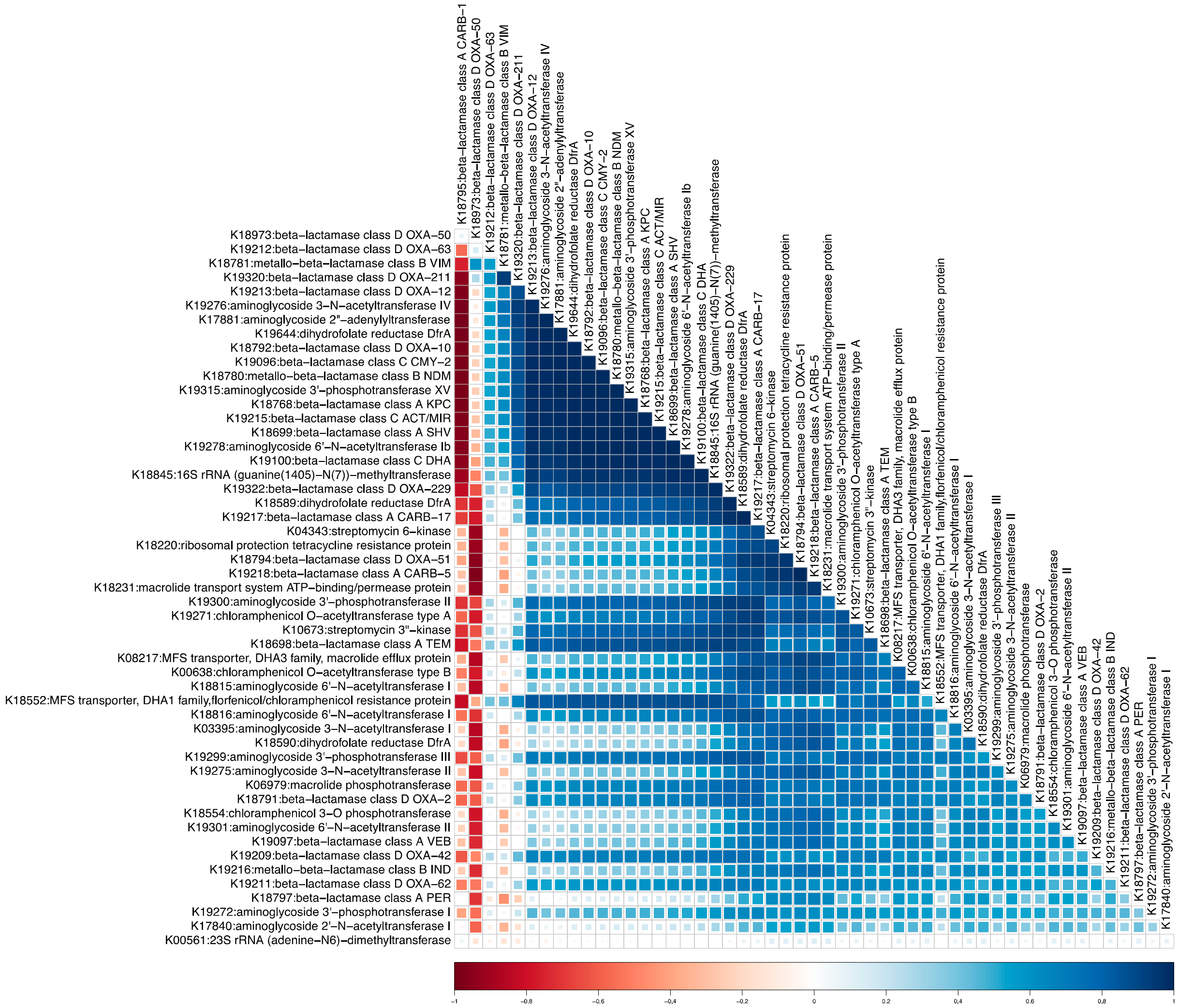

Supplementary Figure S2. Co-occurrence relationship between predictive antimicrobial resistance genes (piARGs) recovered from the residual covariance matrix $\boldsymbol{\Sigma}$ that are not explained by the observed covariates in the GLLVM model in Figure 5. Here, blue represent the positive correlation (increase in the abundance of piARG1 leads to an increase in the abundance of piARG2) and red represent the negative relationship (decrease in the abundance of piARG1 leads to an decrease in the abundance of piARG2).

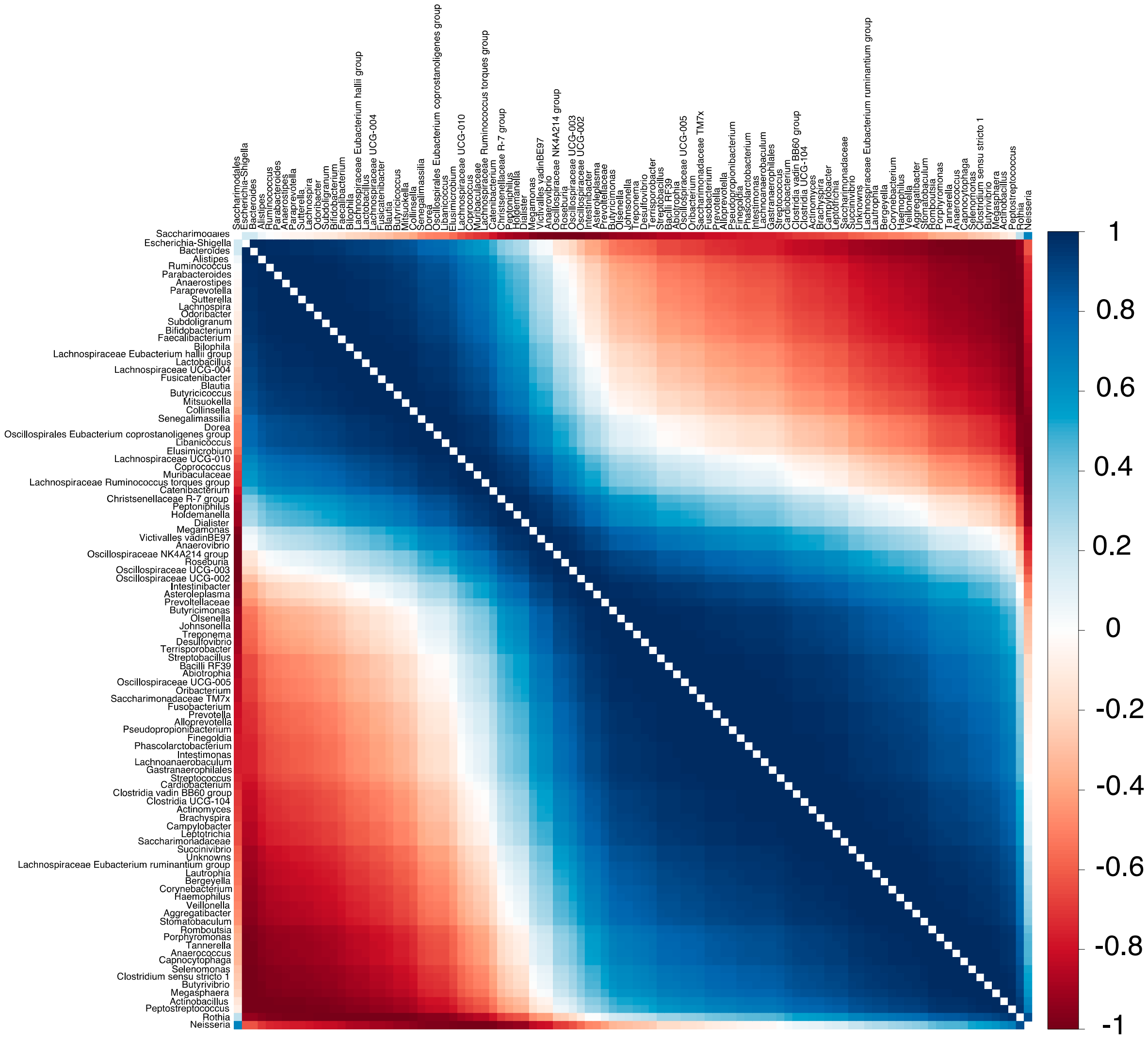

Supplementary Figure S3. **Co-occurrence relationship between microbes** recovered from the residual covariance matrix $\boldsymbol{\Sigma}$ that are not explained by the observed covariates in the GLLVM model in Figure 4. Here, blue represent the positive correlation (increase in the abundance of Taxa1 leads to an increase in the abundance of Taxa 2), and red represent the negative relationship (decrease in the abundance of Taxa1 leads to an decrease in the abundance of Taxa 2).

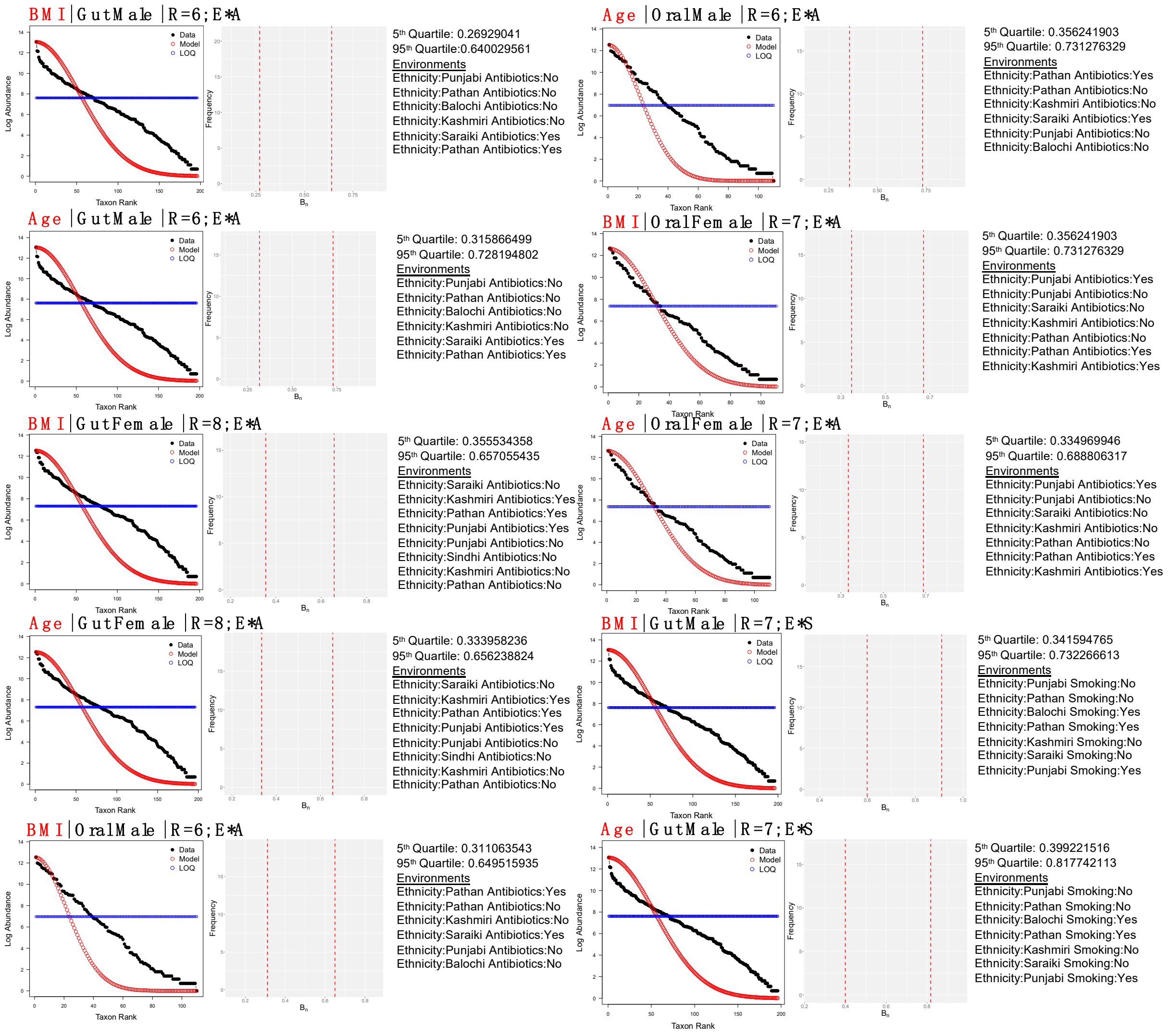

Supplementary Figure S4. We have applied Hurlbert's B_N to two environmental properties, *Age*, and *BMI* considered in this study depending on the expansive set of environments the microbes are observed in, e.g., "*BMI | Gut Male | R = 6; E * A"* represent *BMI* as the environmental property and samples from the male gut with 6 environments as a combination of *Ethnicity* (E) and *Antibiotic status* (A). Similarly, *E * S* represent all possible combinations of *Ethnicity* and *Smoking* status. All the analyses are given as three figure tuples. The left figures represent the rank distribution of the taxa observed in the dataset represented as black. The lognormal rank distribution model is then shown as red circles. The limit of quantification threshold, are then shown as blue circles, is 1.65 standard deviations from zero. Any taxa that fall below the limit of quantification were excluded from the analyses. The middle figure represents the null model distributions generated from applying Hurlbert's B_N calculated from 999 randomly generated taxon distributions. Red dotted lines indicate the fifth and 95th quantiles. Taxa that are high when an environmental property (*Age* or *BMI*) is low have a Hurlbert's B_N below the 5th quantile, and conversely, taxa that are high when the environmental property is high have a Hurlbert's B_N above the 95th quantile of those null models. The right figure represents the values of fifth and 95th quantiles along with the details of all possible environments.

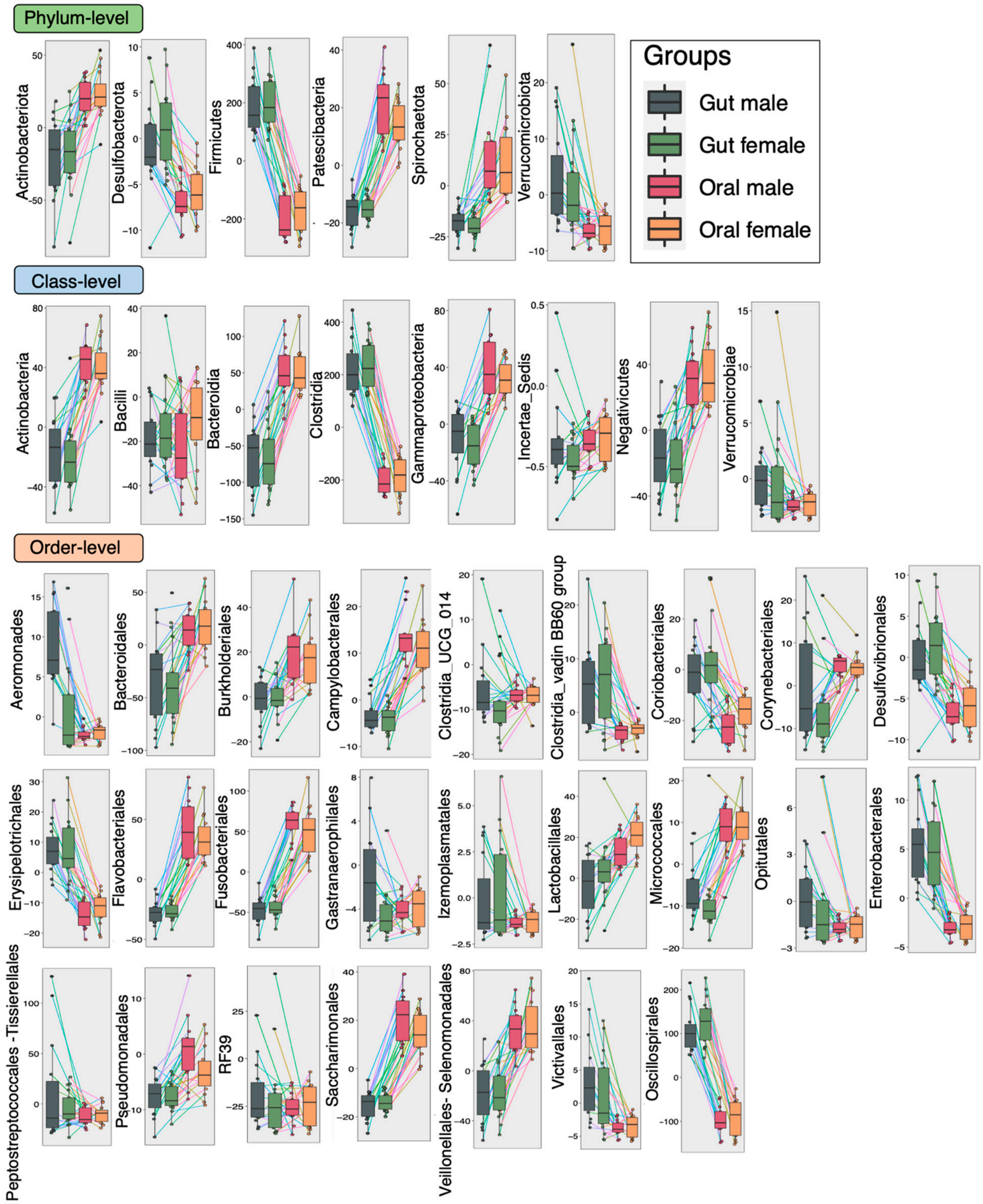

Supplementary Figure S5. **Subset of taxa (at phylum, class, and order level)** that are differentially abundant between the cohorts considered in this study using QCAT-C association test that takes into account paired nature of samples i.e., originating from the same subject connected by lines. The values represent the TSS+CLR normalized abundances of individual taxa.

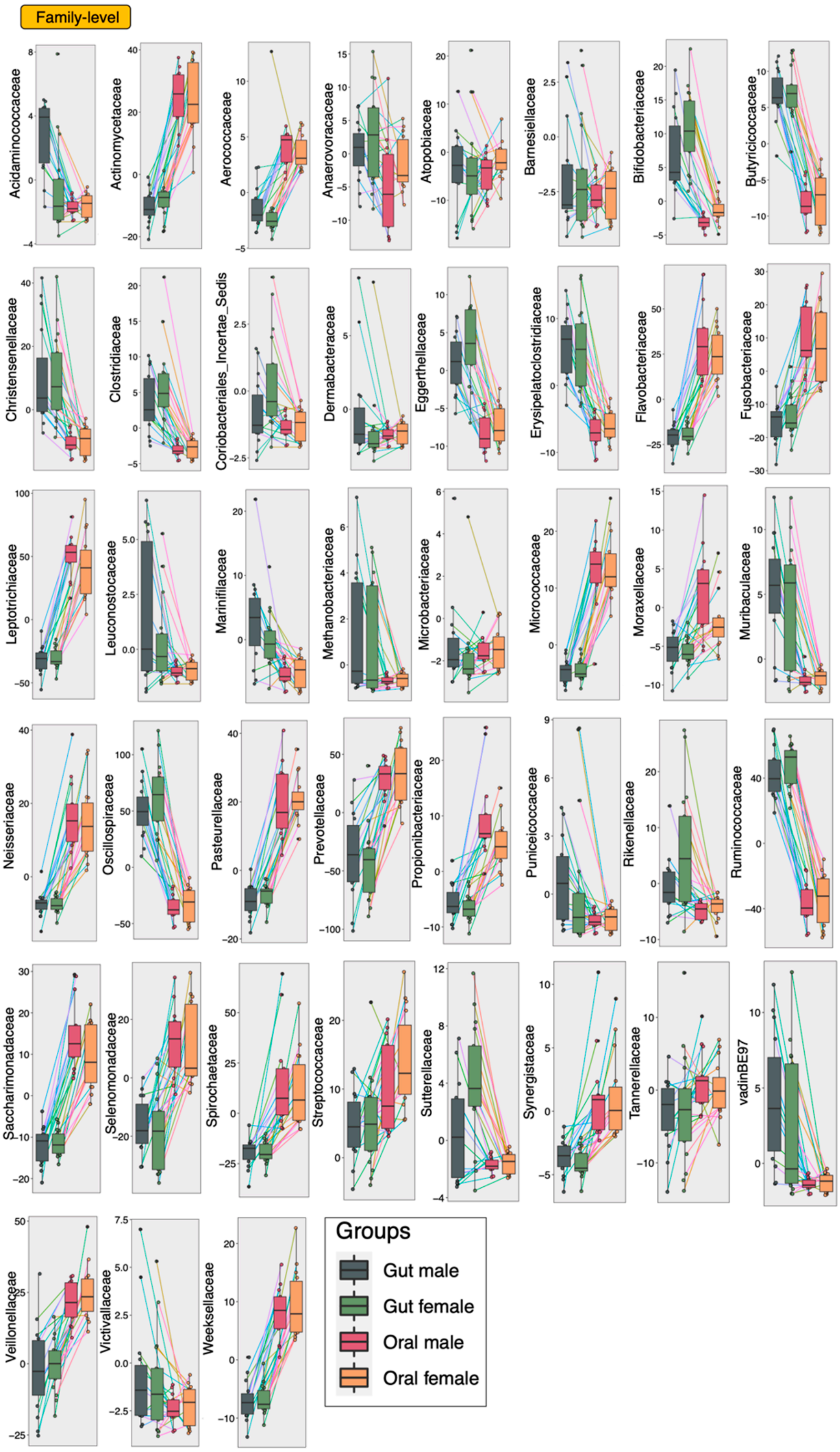

Supplementary Figure S6. **Subset of taxa (at family level)** that are differentially abundant between the cohorts considered in this study using QCAT-C association test that takes into account paired nature of samples i.e., originating from the same subject connected by lines. The values represent the TSS+CLR normalized abundances of individual taxa.

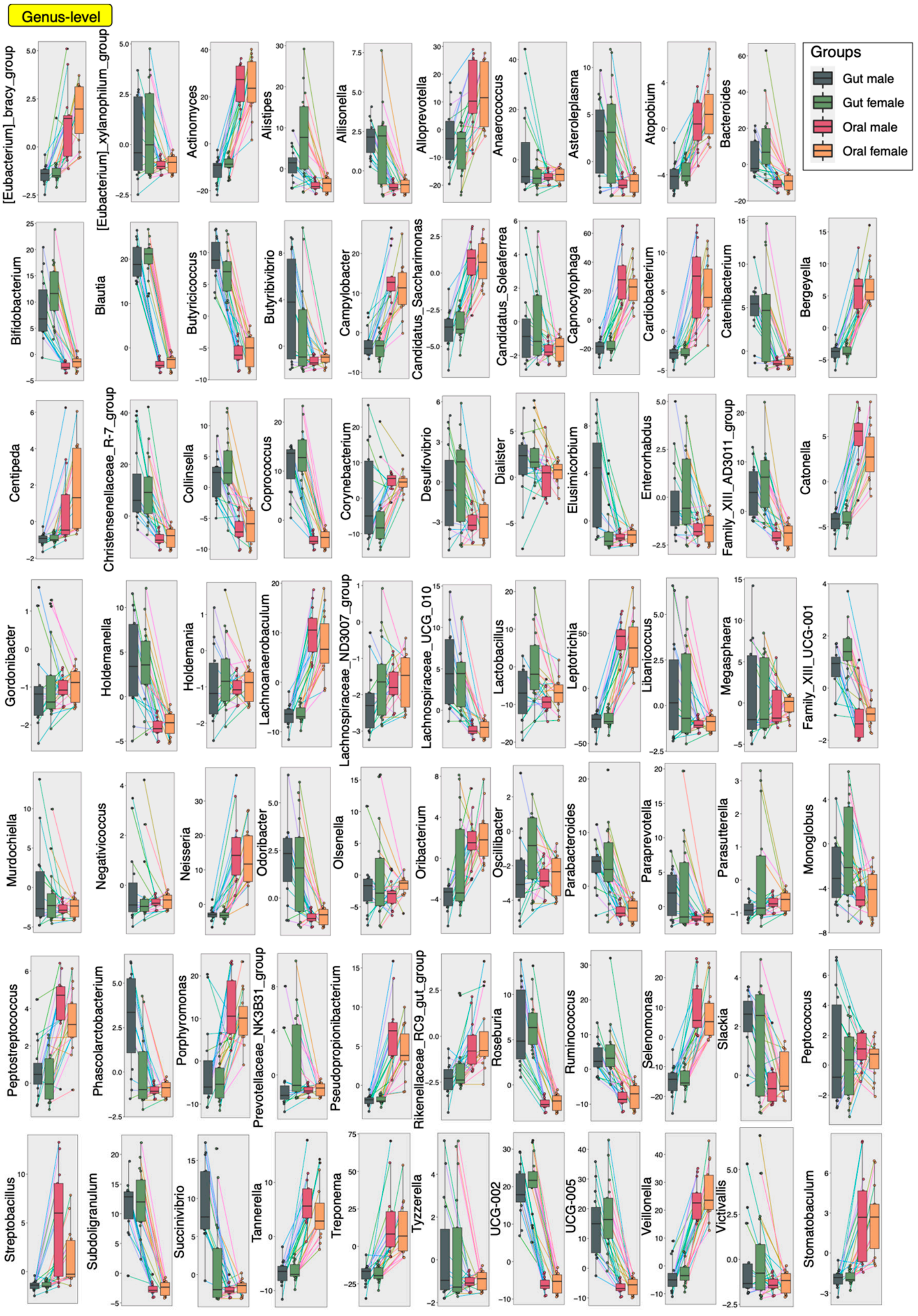

Supplementary Figure S7. **Subset of taxa (at genus level)** that are differentially abundant between the cohorts considered in this study using QCAT-C association test that takes into account paired nature of samples i.e., originating from the same subject connected by lines. The values represent the TSS+CLR normalized abundances of individual taxa.

Supplementary Table S2. Master list of predictive antimicrobial resistance genes (piARGs) from https://www.genome.jp/kegg/annotation/br01600.html. The piARGs that were detected in our study are highlighted in grey along with the annotation information.

Sr No:	Class	KEGG Ortholog	Name	Threat_level	Drug_group
1	A 2b	K18698	beta-lactamase class A TEM	A3 B1 B4 B6 B7 B8 B12	Extended-spectrum cephalosporin (DG01776, DG01777),Monobactam (DG01454)
2	A 2b	K18699	beta-lactamase class A SHV	B1 B4 B5	Extended-spectrum cephalosporin (DG01776, DG01777),Monobactam (DG01454)
3	A 2b	K18767	beta-lactamase class A CTX-M	B1 B4 B7 B8 B9	Extended-spectrum cephalosporin (DG01776, DG01777),Monobactam (DG01454)
4	A 2b	K18797	beta-lactamase class A PER	B1 B4 B6 B7 B8	Extended-spectrum cephalosporin (DG01776, DG01777),Monobactam (DG01454)
5	A 2b	K19097	beta-lactamase class A VEB	B1 B4 B6	Extended-spectrum cephalosporin (DG01776, DG01777)
6	A 2b	K19317	beta-lactamase class A BEL	B6	Extended-spectrum cephalosporin (DG01776, DG01777)
7	A 2b	K18796	beta-lactamase class A LAP		
8	A 2f	K18768	beta-lactamase class A KPC	A2 B1 B6	Carbapenem (DG01458)
9	A 2f	K18970	beta-lactamase class A GES	B1 B4 B6	Extended-spectrum cephalosporin (DG01776, DG01777),Carbapenem (DG01458)
10	A 2f	K19316	beta-lactamase class A IMI/SME	A2	Carbapenem (DG01458)
11	A 2f	K22346	beta-lactamase class A SME	A2	Carbapenem (DG01458) Second-generation cephalosporin (DG01775) Monobactam (DG01454)

12	A 2c	K18795	beta-lactamase class A CARB-1	B6 B7	Carbenicillin (DG00519)
13	A 2c	K19218	beta-lactamase class A CARB-5	B1	Carbenicillin (DG00519)
14	A 2c	K19217	beta-lactamase class A CARB-17		
15	D 2d	K18794	beta-lactamase class D OXA-51	B1	Carbapenem (DG01458)
16	D 2d	K19318	beta-lactamase class D OXA-213	B1	Carbapenem (DG01458)
17	D 2d	K18971	beta-lactamase class D OXA-24	B1	Carbapenem (DG01458)
18	D 2d	K18793	beta-lactamase class D OXA-23	A2 B1	Carbapenem (DG01458)
19	D 2d	K19319	beta-lactamase class D OXA-134	B1	Carbapenem (DG01458)
20	D 2d	K19320	beta-lactamase class D OXA-211	B1	Carbapenem (DG01458)
21	D 2d	K19321	beta-lactamase class D OXA-214	B1	Extended spectrum penicillin (DG01780) Carbapenem (DG01458) (weak)
22	D 2d	K19322	beta-lactamase class D OXA-229	B1	Carbapenem (DG01458)
23	D 2d	K18972	beta-lactamase class D OXA-58	B1	Carbapenem (DG01458)
24	D 2d	K21266	beta-lactamase class D OXA-286	B1	
25	D 2d	K18973	beta-lactamase class D OXA-50	B6	Narrow-spectrum penicillin (DG01779)
26	D 2d	K19211	beta-lactamase class D OXA-62		Carbapenem (DG01458)
27	D 2d	K18791	beta-lactamase class D OXA-2	B1 B4 B6 B7 B8	Extended-spectrum cephalosporin (DG01776, DG01777)
28	D 2d	K18792	beta-lactamase class D OXA-10	B1 B4 B6	Extended-spectrum cephalosporin (DG01776, DG01777)
29	D 2d	K18976	beta-lactamase class D OXA-48	A2	Carbapenem (DG01458
30	D 2d	K19210	beta-lactamase class D OXA-61	B2	Narrow-spectrum penicillin (DG01779)
31	D 2d	K19212	beta-lactamase class D OXA-63		Narrow-spectrum penicillin (DG01779)
32	D 2d	K18790	beta-lactamase class D OXA-1	B4 B6 B7 B9	Extended-spectrum cephalosporin (DG01776, DG01777),Extended spectrum penicillin (DG01780)
33	D 2d	K19098	beta-lactamase class D OXA-9		Narrow-spectrum penicillin (DG01779)
34	D 2d	K19209	beta-lactamase class D OXA-42		Narrow-spectrum penicillin (DG01779)

35	D 2d	K19213	beta-lactamase class D OXA-12		Narrow-spectrum penicillin (DG01779)
36	D 2d	K21276	beta-lactamase class D OXA-22		Narrow-spectrum penicillin (DG01779)
37	D 2d	K21277	beta-lactamase class D OXA-60		Narrow-spectrum penicillin (DG01779),Carbapenem (DG01458) (weak)
38	D 2d	K22331	beta-lactamase class D OXA-184	B2	
39	D 2d	K22332	beta-lactamase class D OXA-548		
40	D 2d	K22333	beta-lactamase class D OXA-493		
41	D 2d	K22334	beta-lactamase class D OXA-464		
42	D 2d	K22335	beta-lactamase class D OXA-114		Extended spectrum penicillin (DG01780),Third-generation cephalosporin (DG01776)
43	D 2d	K22351	beta-lactamase class D OXA-209		Extended-spectrum penicillin (DG01780)
44	D 2d	K22352	beta-lactamase class D OXA-29		Extended-spectrum penicillin (DG01780)
45	C 1	K19095	beta-lactamase class C CMY-1	B4	Extended-spectrum cephalosporin (DG01776, DG01777)
46	C 1	K19096	beta-lactamase class C CMY-2	B4 B7	Second-generation cephalosporin (DG01775),Third-generation cephalosporin (DG01776)
47	C 1	K19100	beta-lactamase class C DHA		
48	C 1	K19101	beta-lactamase class C FOX		
49	C 1	K19214	beta-lactamase class C ACC		
50	C 1	K19215	beta-lactamase class C ACT/MIR		Extended-spectrum penicillin (DG01780),Second-generation cephalosporin (DG01775)
51	C 1	K20319	beta-lactamase class C ADC		
52	C 1	K20320	beta-lactamase class C PDC		

53	B	K18782	metallo-beta-lactamase class B IMP	A2 B1 B4 B6 B9	Extended-spectrum cephalosporin (DG01776, DG01777),Carbapenem (DG01458)
54	B	K18781	metallo-beta-lactamase class B VIM	B4 B6	Extended-spectrum cephalosporin (DG01776, DG01777),Carbapenem (DG01458)
55	B	K18780	metallo-beta-lactamase class B NDM	A2 B1	Carbapenem (DG01458)
56	B	K19099	metallo-beta-lactamase class B GIM	A2 B6	Carbapenem (DG01458)
57	B	K19216	metallo-beta-lactamase class B IND		Carbapenem (DG01458)
58	O	K17840	aminoglycoside 2'-N-acetyltransferase I	B6 B12	Aminoglycoside (DG01447)
59	O	K03395	aminoglycoside 3-N-acetyltransferase I	B1 B6 B7	Aminoglycoside (DG01447)
60	O	K19275	aminoglycoside 3-N-acetyltransferase II	B1 B7	Aminoglycoside (DG01447)
61	O	K19276	aminoglycoside 3-N-acetyltransferase IV	B6	Aminoglycoside (DG01447)
62	O	K19277	aminoglycoside 3-N-acetyltransferase VI	B7	Aminoglycoside (DG01447)
63	O	K19278	aminoglycoside 6'-N-acetyltransferase Ib	B1 B6	Aminoglycoside (DG01447)
64	O	K19301	aminoglycoside 6'-N-acetyltransferase II	B1 B6	Aminoglycoside (DG01447)
65	O	K18815	aminoglycoside 6'-N-acetyltransferase I	B1 B6 B7	Aminoglycoside (DG01447)
66	O	K18816	aminoglycoside 6'-N-acetyltransferase I	B1 B6 B7 B11	Aminoglycoside (DG01447)
67	O	K17881	aminoglycoside 2''-adenylyltransferase	B1	Aminoglycoside (DG01447)

68	O	K19544	aminoglycoside 4'-adenylyltransferase	B6	Aminoglycoside (DG01447)
69	O	K19272	aminoglycoside 3'-phosphotransferase I	B1 B7	Aminoglycoside (DG01447)
70	O	K19300	aminoglycoside 3'-phosphotransferase II	B6	Aminoglycoside (DG01447)
71	O	K19299	aminoglycoside 3'-phosphotransferase III	B11	Aminoglycoside (DG01447)
72	O	K19274	aminoglycoside 3'-phosphotransferase VI	B1	Aminoglycoside (DG01447)
73	O	K19315	aminoglycoside 3'-phosphotransferase XV	B6	Aminoglycoside (DG01447)
74	O	K10673	streptomycin 3''-kinase	B1 B6 B7	Aminoglycoside (DG01447)
75	O	K04343	streptomycin 6-kinase	B1 B6 B7	Aminoglycoside (DG01447)
76	O	K18845	16S rRNA (guanine(1405)-N(7))-methyltransferase	B1 B6	Aminoglycoside (DG01447)
77	O	K18220	ribosomal protection tetracycline resistance protein	A1	Tetracycline (DG00005)
78	O	K00561	23S rRNA (adenine-N6)-dimethyl transferase	B11	Macrolide antibiotic (DG01551)
79	O	K18231	macrolide transport system ATP-binding/permease protein	B1 B11	Macrolide antibiotic (DG01551)
80	O	K06979	macrolide phosphotransferase	B1	Macrolide antibiotic (DG01551)
81	O	K08217	MFS transporter, DHA3 family, macrolide efflux protein	B7 B11	Macrolide antibiotic (DG01551)
82	O	K18552	MFS transporter, DHA1 family, florfenicol/chloramphenicol resistance protein	B6 B7	Phenicol (DG01576)
83	O	K19271	chloramphenicol O-acetyltransferase type A	A3 B1 B6 B7 B11	Phenicol (DG01576)
84	O	K00638	chloramphenicol O-acetyltransferase type B	B1 B6 B7	Phenicol (DG01576)

85	O	K18554	chloramphenicol 3-O phosphotransferase	B12	Phenicol (DG01576)
86	O	K18589	dihydrofolate reductase DfrA	B1 B6 B7	Trimethoprim (DG01581)
87	O	K19643	dihydrofolate reductase DfrA	B1	Trimethoprim (DG01581)
88	O	K18590	dihydrofolate reductase DfrA	B7	Trimethoprim (DG01581)
89	O	K19644	dihydrofolate reductase DfrA	B7	Trimethoprim (DG01581)
90	O	K19645	dihydrofolate reductase DfrB	B6 B7	Trimethoprim (DG01581)
